# Relationship Between Lower-Body Power and Sport-Specific Start Performance in International-Level BMX Riders

**DOI:** 10.3390/jfmk11020198

**Published:** 2026-05-17

**Authors:** Noel Marcen-Cinca, Pablo Jesús Bascuas, Juan Rabal-Pelay

**Affiliations:** 1 Faculty of Health Sciences, Universidad San Jorge, Villanueva de Gállego, 50830 Zaragoza, Spain; pbascuas@usj.es; 2 Valor A Research Group, Health Sciences Faculty, Universidad San Jorge, 50830 Zaragoza, Spain

**Keywords:** BMX, start performance, peak power, wingate test, sprint cycling, timing gates, force–velocity profile, lower-body power

## Abstract

**Background:** Bicycle Motocross (BMX) performance is strongly influenced by the start phase, which requires rapid force and power production to achieve optimal race posi-tioning; however, the relationship between lower-body power and sport-specific start performance remains insufficiently investigated. **Objectives:** The aim of this cross-sectional study was to assess lower-body muscular performance and analyze its relationship with start performance in international BMX riders. **Methods:** Ten international-level BMX riders (*n* = 10) completed a testing battery including squat jump and countermovement jump, force–velocity profile assessment, and a Wingate test preceded by 5 s maximal sprints to determine peak power (PP1, PP2), peak power during the 30 s Wingate test, and mean power. A sport-specific start test was performed on a BMX ramp, with time over the first 15 m recorded using photocell timing gates. **Results:** StartGate 15 m time showed a large negative correlation with PP2 (r = −0.800, 95% CI: −0.95 to −0.33, *p* = 0.05), whereas no significant correlations were observed with vertical jump performance or Power Mean Wingate. Strong correlations were observed among laboratory-based power variables. **Conclusions:** These findings suggest that short-duration peak cycling power may be associated with BMX start performance. However, given the small sample size and the borderline *p*-value, this relationship should be interpreted with caution. Sport-specific start testing may provide relevant information for performance assessment and training monitoring in international BMX riders.

## 1. Introduction

Bicycle Motocross (BMX) is an Olympic cycling discipline in which riders compete in individual qualification races followed by successive heats involving up to eight riders. Races are held on tracks 300–400 m long [1,2,3,4] that combine dirt and asphalt surfaces and include an elevated start gate (5–8 m high), jumps, pumps and turns [4,5]. Each race lasts approximately 30–50 s, with recovery periods of 15–30 min between heats during official competitions [6].

BMX performance depends on both technical skills and physical capacities, with a high demand on phosphagen and glycolytic energy systems [7]. Although sprint ability is important across cycling disciplines [8], it is particularly decisive in BMX. The capacity to generate high mechanical power during pedalling, driven by neuromuscular and musculoskeletal function, is a key determinant of performance. Riders must produce high force at high contraction velocities, especially during the initial phase of the race. Indeed, the first 8–9 s are critical for achieving a favourable position before the first obstacle, highlighting the importance of the start [9,10].

Elite BMX riders exhibit high levels of neuromuscular strength as well as great jumping ability. Petruolo et al. [11] reported that elite BMX cyclists achieved peak power outputs of 1498 ± 189 W during a 5 s sprint test and 1599 ± 265 W during a Wingate test, with a mean Wingate power of 1344 ± 158 W. In addition, countermovement jump peak power reached 4624 ± 768 W. These values confirm the highly anaerobic and explosive profile of this population and exceed those typically observed in endurance-oriented cycling disciplines. Furthermore, maximal squat strength (1 RM) and force–velocity profile-derived maximal power have shown strong associations with sprint peak power, Wingate performance (30 s of pedalling developing the maximum possible effort), and track performance [8]. Jumping performance, assessed through squat jump and countermovement jump, has also been negatively correlated with BMX track competition time in elite riders [12].

Despite the recognized importance of the start as a conditional, technical, and tactical determinant of BMX performance, no previous research has specifically examined sprint capacity from the starting gate. Given that the initial acceleration phase strongly influences race positioning and overall outcome, the assessment of lower-limb neuromuscular performance in relation to start performance appears warranted. From a practical perspective, coaches and athletes require objective tools to monitor performance and optimize training strategies.

Therefore, the primary aim of this study was to assess lower-body muscular performance in international-level BMX riders. A secondary aim was to analyze the relationship between strength outputs and performance time following the start ramp.

Based on the neuromuscular demands of the BMX start and the previously reported associations between maximal strength, power output, and sprint performance, we hypothesized that (i) international-level BMX riders would exhibit high levels of lower-body maximal strength and power, and (ii) greater strength and power outputs (e.g., 1 RM squat, force–velocity profile-derived maximal power, and sprint/Wingate peak power) would be significantly associated with shorter times during the start ramp phase.

## 2. Materials and Methods

The design was a cross-sectional observational study. Tests were chosen following recommendations of the research literature [7,11,13] to assess the force–velocity profile, pedal power, jump power and its application to a field test. This study was held in an international venue, in the 4 days following a BMX European Cup. On the first day after the competition day, subjects and coaches had the information about the procedure. Tests were carried out on the second, third and fourth day after the competition during morning hours (between 10.00 h and 13.00 h).

This battery test allows characterization of the subject’s physical condition in a moment of good physical shape and may permit to establishing relationships between different conditional factors and sport-specific determinants.

### 2.1. Participants

A total of 10 subjects included in junior and elite categories participated in this study. To be included in the study, participants were required to be active BMX riders with experience competing at the international level. Participants were excluded if they had any current musculoskeletal injury or medical condition that could impair performance or prevent them from completing the testing protocol safely. All participants were injury-free at the time of testing. Participants were recruited from 5 different national teams. All of them resided in the same venue during the evaluation process. All BMX riders were classified as Tier 4 (International Level) [14]. The protocol was approved by the Ethics Research Committee of a regional government. Every procedure was conducted in accordance with the principles of the Declaration of Helsinki. Each participant was informed of the nature of the study, the voluntariness of the participation and of any potential adverse effects and signed an informed consent form.

### 2.2. Procedures and Data Collection

On the first day, anthropometric data and performance data were collected to characterize subjects: height, weight, body mass index (BMI), and leg length. Two measures (1: trochanter–tiptoe, and 2: in squat position, knees in 90°, distance from trochanter to floor) were measured. The athlete’s gender, category, and level (ranking) were registered. The squat jump (SJ) test was the first to be performed. Each athlete had three attempts, and the best one was used for the subsequent analysis. A test to study the force–velocity profile of athletes was performed following recommendations of Morin [15,16,17]. This approach is used to characterize the neuromuscular system and its ability to generate force. Following the general warming up, athletes were asked to perform a submaximal countermovement jump (around 70% of their best) and then 3 maximal jumps were performed under 3 conditions: (1) a regular counter movement jump (CMJ), (2) a CMJ with 20% of body weight added and (3) a CMJ with 40% of body weight. The best jump of each condition was registered. Force–velocity outcomes (Table 1) and force–velocity imbalance were calculated and analyzed. Jumps were registered with the OptogaitTM System (Microgate, Bolzano, Italy) and Optogait software 1.12.1.0 (Microgate, Bolzano, Italy).

On the second day, participants performed a Wingate test preceded by a standardized warm-up and sprint protocol on a Wattbike ergometer (WattBike Atom, WattBike Ltd., Nottingham, UK). The protocol consisted of 10 min of low-intensity pedalling, followed by a 5 s effort at approximately 70% of perceived maximal effort, 1 min of low-intensity pedalling, and a 5 s maximal sprint against resistance level 1 of the ergometer (Sprint 1). After 1 min of passive recovery, participants performed a second 5 s maximal sprint against the resistance level automatically determined by the ergometer according to body mass (Sprint 2) [20]. Following 5 min of passive recovery, participants completed a 30 s maximal Wingate test. The Wingate test consists of a 30 s maximal sprint, which is widely used in cycling [21] and it has been already applied in BMX [11]. The objective was to measure the ability of the glycolytic and phosphagen systems, by measuring the power generated in a cyclo-ergometer. The ergometer shows good validity and reliability and has been used in other studies [22,23,24]. The following variables were recorded: (1) peak power in Sprint 1 (PP1, W); (2) peak power in Sprint 2 absolute (PP2, W) and relative to body weight (Power Sprint, W/kg); (3) peak power during the 30 s Wingate test (Power Peak Wingate, W; (4) mean power during the 30 s Wingate test (Mean Power Wingate, W); (5) maximal cadence in Sprint 1 (MaxCad1, rpm); (6) maximal cadence in Sprint 2 (MaxCad2, rpm); and (7) mean cadence during the 30-s Wingate test (Mean Cadence Wingate, rpm).

Finally, on the third day, a sport-specific test was proposed: a start-test in a 5 m ramp. The warm-up preceding the field test was conducted by each team’s coaching staff. Although this reflects real-world practice, it may have introduced some degree of variability between athletes. A total of 3 starts were performed by each athlete; instructions were to start as if they were in a competition. A photocell timing gate (ProStart gate and laser sensors. ProStart, Sainte Soulle, France) was placed at 15 m. The fastest trial was registered.

### 2.3. Statistical Analysis

All statistical procedures were completed on IBM™ SPSS™ Statistics (version 24, IBM Corporation, Somers, NY, USA). Descriptive statistics (mean ± standard deviation “SD”) were calculated. The normality of the data was assessed using the Shapiro–Wilk test, and all variables showed a normal distribution. Therefore, parametric tests were applied for all analyses. Differences between groups were analyzed using independent *t*-test. The magnitude of each change was assessed using Cohen d effect size [25] (ES; d ≤ 0.2, small; 0.5–0.79, moderate; ≥0.8, strong). Pearson correlation coefficients were calculated to determine associations between variables. The 95% confidence interval was reported for the main significant correlation. Pearson’s r value ranging between 0.5 and 0.7 was considered as moderate correlation: 0.7–0.9 as strong correlation; values higher than 0.9 as very strong correlation. Significance level was set at *p* ≤ 0.05.

## 3. Results

The statistical analysis showed differences in age and height between junior and elite cyclists. No differences were found in body mass or BMI between categories (Table 2).

Results of the battery test are shown in Table 3. Performance in the sport-specific start test (StartGate 15 m) showed a selective pattern of associations with conditional performance variables (Table 4). The time required to cover the first 15 m after the BMX start ramp was strongly correlated with peak power achieved during the second 5 s sprint effort of the protocol (PP2; r = −0.80, 95% CI: −0.95 to −0.33, *p* = 0.05, R^2^ = 0.64). Although the *p*-value reached the conventional threshold for statistical significance, the large effect size indicates that PP2 explained approximately 64% of the variance in start performance, supporting the practical relevance of this association.

No significant correlations were observed between StartGate 15 m time and vertical jump performance variables (SJ or CMJ), and no significant correlation was found with mean power output during the 30-s Wingate test (Power Mean Wingate).

Strong correlations were observed among laboratory-based neuromuscular performance variables. Vertical jump performance (SJ and CMJ) was strongly associated with cycling power outcomes, including peak power during the Wingate test (Power Peak Wingate) and relative mean Wingate power (W·kg^−1^). Additionally, PP2 showed strong correlation with other cycling power variables such as Power Peak Wingate and Relative Wingate power, indicating internal consistency among laboratory measures of short-duration power output.

However, these laboratory-based neuromuscular variables did not translate uniformly to sport-specific start performance, as evidenced by the absence of significant correlations between jump performance and StartGate 15 m time.

## 4. Discussion

The main finding of the present study is that performance in a sport-specific field test assessing the BMX start (StartGate 15 m) was significantly associated with short-duration peak cycling power (PP2), while showing no relationship with vertical jump performance. This result highlights the relevance of ecologically valid field-based assessments when identifying performance determinants in BMX racing.

Previous research has identified the start phase as one of the most critical moments of a BMX race, given its influence on rider positioning before the first obstacle and its subsequent impact on race outcome [6,26,27]. However, most studies have relied on laboratory or generalized neuromuscular tests, with limited use of sport-specific start performance measures. The present study extends existing literature by directly linking a timed BMX ramp start test with laboratory power variables.

The strong negative correlation observed between StartGate 15 m time and PP2 indicates that riders capable of producing higher peak power during very short sprint efforts were able to accelerate more effectively during the start phase. This finding is consistent with previous work describing the BMX gate start as a phase characterized by maximal force and power application under strict temporal constraints [6]. Sprint 1 (PP1) was performed using the lowest resistance setting, which likely resulted in a reduced torque–cadence relationship and may explain its lack of association with start performance. In contrast, PP2 was conducted under body-mass-adjusted resistance conditions that better reflect the mechanical demands of the start, supporting the notion that performance assessments should be carried out using sport-specific equipment and loading conditions.

Rylands et al. demonstrated that the timing of power production, rather than mean power output, is a key determinant of BMX start effectiveness, with higher field-based peak power and shorter time to peak power observed in real track conditions compared to laboratory testing [26,27]. The present results align with this perspective, showing that a short-duration peak power variable (PP2) is more strongly related to start performance than longer-duration measures such as mean Wingate power.

Contrary to previous studies reporting associations between vertical jump performance and overall BMX race time or general performance indices [8,12], no significant relationships were observed between SJ or CMJ height and StartGate 15 m time in the present study. This discrepancy may be explained by the specific mechanical and coordinative demands of the BMX start.

While vertical jump tests are widely used to assess lower-body explosive strength, they predominantly reflect vertical force production in a simplified motor task. In contrast, the BMX start requires rapid horizontal force application, precise coordination between upper and lower body, and effective interaction with the bicycle during gate release and ramp descent [6,28]. As such, vertical jump performance may represent a general neuromuscular quality that does not directly translate to start-specific acceleration capacity.

From a practical standpoint, the observed correlation (r = −0.80, R^2^ = 0.64) indicates that PP2 explained approximately 64% of the variance in StartGate 15 m time. While this represents a substantial proportion, approximately 36% of the variance remains unexplained and is likely attributable to factors not captured by the present testing battery, such as technical skill during gate release and ramp descent, reaction time at the start signal, pedalling technique, gear selection strategy, and upper-body coordination during the initial acceleration phase. Future studies should incorporate these variables to develop a more comprehensive model of BMX start performance.

The present findings support previous calls for greater ecological validity in BMX performance assessment. Several studies have reported substantial differences between laboratory and field-based measures of power output in BMX riders, with higher peak power values and shorter times to peak power observed in real track conditions [26,29].

Daneshfar et al. emphasized the importance of field-based power analysis in BMX, noting that laboratory tests may fail to capture the complex interaction between rider, bicycle, and track [7]. The selective association observed in the present study—where only short-duration peak power correlated with a field-based start test—reinforces the need to integrate sport-specific assessments when evaluating BMX performance.

Regarding the values found in the F–V profile of the present study, they were different from those reported in other studies of BMX riders [8,13]. Mean of junior-elite F_0_ is lower than reporter in Moya-Ramón et al. and Gross et al. (2514 N vs. 3126 N [8]/3067 N [13]. In contrast, Pmax (3107 W vs. 1947 W/1935 W) and V_0_ (4.98 m·s^−1^ vs. 2.6 m·s^−1^/2.5 m·s^−1^) were higher. These differences may be explained by the type of jump used during the tests. In the present study, a countermovement jump was used, while their studies used a squat jump without countermovement. The observed differences in force, velocity, and power outcomes can be primarily explained by the biomechanics of the jumping tasks. While the squat jump involves only a concentric (propulsive) phase, the countermovement jump includes a coupled eccentric–concentric action with utilization of the stretch–shortening cycle. Consequently, CMJ-based assessments typically reflect higher power output and V_0_ values, together with lower F_0_ values, compared with those derived from squat jumps. Nevertheless, despite these biomechanical differences, the force–velocity and load–velocity variables obtained from the jumping assessments did not correlate with 15 m start time performance in the present study. The Pmax of jump was similar to that reported in professional rugby players in CMJ F–V profile (3101 W) [30].

Except for theoretical maximal force (F_0_), none of the other variables of the CMJ jump force–velocity relationship differed between the elite and junior groups. These findings suggest that these variables may not be sensitive to performance differences in cycling sprint capacity. In the study by Gross et al. [13], a strong correlation was observed between F0 (R = 0.77) and maximal jump power (R = 0.85) with pedaling power during the start of the test. Moya-Ramón et al. [8] also found an association between maximal jump power and peak cycling power (W) (R = 0.85), as well as between F_0_ and peak cycling power (R = 0.87). In contrast, in the present investigation, none of the jump force–velocity relationship metrics correlated with power output in the Wattbike tests or with the start-ramp time. In line with the systematic review by Becerra-Patiño et al. [4], the findings of the present study highlight that real-world evaluations provide a more accurate indication of performance than laboratory-based tests.

Taken together, these results suggest that BMX performance profiling should distinguish between general neuromuscular capacity and start-specific performance ability. While vertical jump tests and longer-duration cycling tests provide valuable information about an athlete’s overall physical profile, they may not be sufficient to predict start performance, which appears to depend more strongly on rapid power production and acceleration capacity [6,27]. This interpretation is consistent with findings showing that BMX riders spend a limited proportion of race time pedalling, with performance heavily influenced by short, high-intensity actions during key phases such as the start and first straight [6,28].

Some methodological limitations should be acknowledged. The sample size was limited, particularly in the elite subgroup, which may have reduced statistical power. However, similar constraints are common in BMX research involving international-level athletes [11,27]. Additionally, junior and elite riders were pooled into a single sample to maximize statistical power. Although both groups compete under equivalent race conditions and were all classified as Tier 4 international-level athletes, this approach may introduce between-group variability that should be considered when interpreting the results. Furthermore, with a sample of 10 participants, the minimum Pearson correlation coefficient detectable at *p* ≤ 0.05 (two-tailed) is approximately r = 0.63, meaning that only large effects could reach statistical significance. Consequently, the non-significant correlations observed in this study should not be interpreted as evidence of absence of association, as the limited statistical power substantially increases the risk of Type II errors for moderate or small effect sizes. Only bivariate correlational analyses were performed, and future studies with larger samples should explore multivariate approaches to better determine the combined explanatory power of these variables. Moreover, the StartGate 15-m test focuses exclusively on the initial acceleration phase and does not account for subsequent technical execution; nevertheless, this phase has been consistently identified as a decisive determinant of BMX performance [6,26].

The results of this study suggest that coaches should incorporate sport-specific start tests, such as the StartGate 15-m test, into their performance monitoring protocols. This test provides a direct and ecologically valid measure of a rider’s ability to accelerate from the start ramp, which is critical for achieving optimal race positioning. Field-based tests such as the StartGate 15-m are particularly valuable because they allow riders to perform truly maximal efforts under realistic technical and motivational conditions, which may be difficult to replicate in laboratory environments. Performing maximal efforts on an actual BMX start ramp enables riders to apply force and coordinate movement in a context that closely resembles competition, potentially resulting in more accurate assessments of performance capacity. The strong relationship observed between start performance and short-duration peak cycling power indicates that training programs should emphasize the development of maximal power output over very short time intervals, particularly through sprint-specific cycling exercises and start-focused drills. In contrast, vertical jump tests may be useful for general neuromuscular assessment but may not accurately reflect start-specific performance. Therefore, combining BMX-specific start assessments with short sprint power testing may help coaches better evaluate performance capabilities, monitor training adaptations, and design more effective, targeted training strategies.

## 5. Conclusions

The present study demonstrates that a sport-specific BMX start test provides unique information about performance that is not captured by traditional laboratory or jump-based assessments. The association between StartGate 15-m time and short-duration peak cycling power, assessed under resistance conditions relative to body mass, highlights the relevance of rapid power production for effective start performance. In contrast, vertical jump performance and force–velocity profile variables were not related to start outcomes. These findings emphasize the importance of task specificity and ecological validity when assessing performance determinants in BMX racing. While force–velocity profiling provides valuable insight into general neuromuscular characteristics, it does not appear to directly predict start ramp performance in this population.

Future research should aim to confirm these findings in larger samples, explore longitudinal adaptations to start-specific training, and incorporate additional performance determinants such as reaction time and technical execution at the start gate.

## Figures and Tables

**Table 1:** Description of force–velocity countermovement jump relationship outcomes [18,19].

Outcomes	Description
F_0_ (N)	Theoretical maximal vertical force (intercept) production extrapolated from the linear loaded countermovement jump Fe relationship
V_0_ (m·s^−1^)	Theoretical maximal movement velocity (intercept) extrapolated from the linear loaded countermovement jump F–V relationship
PMAX (W)	Maximal mechanical external power output in the vertical direction (Pmax = F_0_ × V_0_/4)
Sfv (N·s·m^−1^)	Slope of the force–velocity relationship
Imbalance F–V	F–V imbalance is the percent difference between actual unloaded jump height and maximal jump height than the athlete could achieve corresponding to optimal F–V relationship.

**Table 2:** Baseline characteristics of male BMX riders recruited.

	Junior (*n* = 7)		Elite (*n* = 3)	
Outcomes	Mean	SD	Mean	SD
Age (years)	18.1 *	±2.1	25.3 *	±3.8
Height (cm)	182.4 *	±6.0	172 *	±6.5
Weight (kg)	73.1	±6.7	73	±7.8
BMI (kg·m^−2^)	22.1	±2.5	24.6	±1.3

Standard deviation (SD). * *p* ≤ 0.05 T-Student test between groups. BMI: body mass index.

**Table 3:** Descriptive data of the conditional test performed by BMX riders.

	Junior (*n* = 7)		Elite (*n* = 3)		Effect Size
Outcomes	Mean	SD	Mean	SD	Cohen’s *d*
**Jumps**					
SJ (cm)	42.8	±6.7	47.4	±7.5	0.67
CMJ (cm)	49.3	±4.9	53.1	±4.5	0.90
**F–V Jump Profile**					
F–V imbalance	52	±26	45	±2	0.30
F_0_ (N)	2388 *	±245	2724 *	±136	1.55
V_0_ (m·s^−1^)	5.26	±1.24	4.53	±0.28	0.71
PMax (W)	3126	±776	3076	±52.3	0.07
Sfv (N·s·m^−1^)	−488	±188	−604	±70.2	0.73
**Cycling Test**					
PP1 (W)	1653	±177	1764	±153	0.80
MaxCad1 (rpm)	180	±13	188	±10	0.76
PP2 (W)	1656	±192	1828	±139	1.05
MaxCad2 (rpm)	170 *	±3	180 *	±3	2.09
Power Sprint (W·kg^−1^)	22.8	±0.5	25.1	±1.9	1.36
Power Peak Wingate (W)	1615	±203	1743	±140	0.80
Power Mean Wingate (W)	996	±101	960	±9	0.01
Mean Cadence Wingate	152	±14	162	±19	0.83
StartGate 15 m (s)	2.22	±0.1	2.16	±0.07	0.79

Descriptive data of neuromuscular, force–velocity profile, cycling power, and start performance variables in junior and elite BMX riders. Values are presented as mean ± standard deviation (SD). Effect size is expressed as Cohen’s *d*. SJ: squat jump; CMJ: countermovement jump; F–V: force–velocity; F_0_: theoretical maximal force; V_0_: theoretical maximal velocity; PMax: maximal power; Sfv: slope of the force–velocity relationship; PP1: peak power during Sprint 1; PP2: peak power during Sprint 2; Power Sprint: Power peak during sprint 2 relate to body weight; Power Peak Wingate: peak power during the 30 s Wingate test; Power Mean Wingate: mean power during the 30 s Wingate test; MaxCad1: maximal cadence during Sprint 1; MaxCad2: maximal cadence during Sprint 2; Mean Cadence Wingate: mean cadence during the 30 s Wingate test; StartGate 15 m: time to reach 15 m from the BMX start gate. * *p* ≤ 0.05 between groups.

**Table 4:** Correlation between conditional performance outcomes in junior and elite male BMX riders (*n* = 10).

Outcomes	CMJ	Power Peak Wingate (W)	Power Mean Wingate (W)	Relative Mean Wingate Power (W·kg^−1^)	Power Sprint (W·kg^−1^)	StartGate 15 m (s)
**Jumps**						
SJ	0.91 (*p* < 0.001) *	0.85 (*p* = 0.015) *	0.22 (*p* = 0.631)	0.91 (*p* = 0.011) *	0.91 (*p* = 0.011)	0.32 (*p* = 0.438)
CMJ		0.89 (*p* = 0.007) *	0.40 (*p* = 0.379)	0.94 (*p* = 0.005) *	0.94 (*p* = 0.005)	0.53 (*p* = 0.175)
F_0_ (N)	0.06 (*p* = 0.889)	0.50 (*p* = 0.221)	−0.30 (*p* = 0.567)	0.21 (*p* = 0.730)	0.22 (*p* = 0.642)	−0.03 (*p* = 0.963)
V_0_ (m·s^−1^)	0.27 (*p* = 0.513)	−0.01 (*p* = 0.992)	−0.26 (*p* = 0.622)	0.66 (*p* = 0.227)	0.20 (*p* = 0.666)	0.24 (*p* = 0.699)
PMax (W)	0.34 (*p* = 0.404)	0.29 (*p* = 0.524)	−0.42 (*p* = 0.410)	0.79 (*p* = 0.110)	0.34 (*p* = 0.452)	0.36 (*p* = 0.549)
**Cycling Test**						
PP1	0.49 (*p* = 0.213)	0.96 * (*p* = 0.001)	0.78 * (*p* = 0.023)	0.74 (*p* = 0.058)	0.46 (*p* = 0.217)	−0.88 (*p* = 0.051)
MaxCad1	0.61 (*p* = 0.112)	0.48 (*p* = 0.223)	−0.01 (*p* = 0.977)	0.14 (*p* = 0.770)	−0.26 (*p* = 0.493)	−0.34 (*p* = 0.579)
PP2	0.53 (*p* = 0.175)	0.96 (*p* < 0.001) *	0.68 (*p* = 0.062)	0.76 (*p* = 0.045) *	0.59 (*p* = 0.093)	−0.80 (*p* = 0.050) *
MaxCad2	0.12 (*p* = 0.791)	0.27 (*p* = 0.565)	0.15 (*p* = 0.743)	0.30 (*p* = 0.567)	0.72 * (*p* = 0.043)	−0.14 (*p* = 0.817)
Mean Cadence Wingate	0.52 (*p* = 0.229)	0.58 (*p* = 0.132)	0.01 (*p* = 0.973)	0.38 (*p* = 0.401)	0.23 (*p* = 0.581)	0.74 (*p* = 0.255)

Pearson r (bivariate correlations). SJ: squat jump; CMJ: countermovement jump; F_0_: theoretical maximal force; V_0_: theoretical maximal velocity; PMax: maximal power; PP1: peak power during sprint 1; PP2: peak power during Sprint 2; Power Sprint: Power peak during sprint 2 relate to body weight; Power Peak Wingate: peak power during the 30 s Wingate test; Power Mean Wingate: mean power during the 30 s Wingate test (absolute); Relative Mean Wingate Power: mean power during the 30 s Wingate test relative to body mass; MaxCad1: maximal cadence during Sprint 1; MaxCad2: maximal cadence during Sprint 2; Mean Cadence Wingate: mean cadence during the 30 s Wingate test StartGate 15 m: time to reach 15 m from the BMX start gate. * *p* ≤ 0.05.

## Data Availability

The data presented in this study are openly available in Zenodo at https://doi.org/10.5281/zenodo.18785965.
